# Bone Mineral Densitometry Findings of Children with Newly Diagnosed Celiac Disease

**DOI:** 10.4274/MIRT.6

**Published:** 2011-08-01

**Authors:** Tansel Ansal Balcı, Zehra Pınar Koç, Hüseyin Aydın Mitil

**Affiliations:** 1 Firat University Faculty of Medicine, Nuclear Medicine Department, Elazig, Turkey

**Keywords:** celiac disease, osteoporosis, bone mineral density, age

## Abstract

**Objective:** The effect of Celiac Disease (CD) on children’s bone is the decrease in bone mineral density (BMD). Osteoporosis is a consequence of this decrease and usually manifests in adult ages. Studies in CD patients generally show that bone density of these patients can be different at the same ages for the same duration of disease. The aim of this study is to investigate the relationship between age and bone mineral density of CD patients at first diagnosis.

**Material and Methods:** Ninety one patients (M/F: 36/55; age range: 3-16; mean age: 9.6±3.5) with diagnosis of CD were included in the study. BMD survey from L1-L4 lumbar spine and total hip of the patients was evaluated at presentation. We evaluated the patients in 3 groups according to their ages: Group 1: pre-school (3-7 years old), Group 2: elementary school (8-11 years old) and Group 3: adolescent (12-16 years old). Results were compared using Student’s t test and correlation analysis.

**Results:** The mean disease duration of the patients was 16.4±16.3 months. Mean height and weight of the patients were 124.8±17.9 cm and 27±9.3 kg, respectively and height and weight of 37 patients were in ≤ 3. percentile according to age. The BMD values of both lumbar spine and total hip and Z-scores of lumbar region were in mild correlation with age (r>0.5). There was significant difference between mean ages of patients with low bone mass for chronological age and normal bone densitometry values (p<0.05). There were 27, 36 and 28 patients in Group 1, Group 2 and Group 3, respectively. The difference between mean BMD values of these groups were statistically significant (p<0.05). The mean values of lumbar Z- scores of patients were -1.08±1.27, -1.42±1, -1.86±1.14, respectively for these three groups.

**Conclusion:** Bone mineral densities of CD patients in childhood were lower in elder children at the time of diagnosis. This confirms the opinion that the diagnosis at earlier age results better treatment chance before bone mineral loss appears in CD patients.

**Conflict of interest:**None declared.

## INTRODUCTION

Celiac Disease (CD) is a common inflammatory enteropathy that causes some lifelong complications. One of these complications is osteoporosis and it is associated with increased fracture risk. Patients with CD are generally asymptomatic (1) and osteoporosis and related complications manifest at adult ages (2,3). The important point about CD related osteoporosis is that complete recovery of bone mineral density (BMD) with gluten free diet (GFD) therapy in children is possible but that is not true for adults (4,5). This makes early diagnosis and treatment of these patients necessary. Although most of CD patients have mild symptoms, the loss of bone mineral content of those patients with mild symptoms is fortunately mild, too (6). The fracture risk of symptomatic CD patients was higher than control group but it was not as high as in asymptomatic patients (6). Although we clearly know that CD patients have lower BMD values than normal population in adulthood, it is not known at which ages this decrease occurs. We aimed to evaluate the trend of BMD alteration of CD patients in childhood before any treatment according to age groups. 

## MATERIALS AND METHODS

**Patients**

Ninety one children (36 M, 55 F; age range: 3-16 years; mean: 9.6±3.5 years) with CD referred for BMD measurement between November 2006 and May 2009 were retrospectively evaluated. The patients were newly diagnosed as CD and untreated prior to the BMD measurement. We classified patients into three age groups. Group 1: pre-school age, Group 2: elementary school age and Group 3: adolescent age. 

**Bone Density Measurement**

The BMD was measured by dual energy x-ray absorptiometry method (DXA) (LUNAR DPX, Lunar Corp.) from L1-L4 lumbar and total hip regions. BMD values of these regions and Z-score of L1-L4 lumbar region were obtained. Z-score is the number of standard deviations at system database by which patient's bone density differed from the healthy age matched mean. Z-score ≤-2 was considered low bone mass for chronological age and Z-score >-2 as normal.

The obtained mean value of precision values of our device was: 0.98±0.015. 

**Statistical Analysis**

The results were compared using Student’s t test and the correlation analysis was also performed. P<0.05 was considered statistically significant. The analysis was performed with SPSS version 14.

## RESULTS

Mean disease duration was 16.4±16.3 months according to the anamnesis. Mean height and weight of the patients were 124.8±17.9 cm and 27±9.3 kg, respectively, and height and weight of 37 patients (40.7%) were in ≤ 3. percentile according to age. 

**Figure 1:**
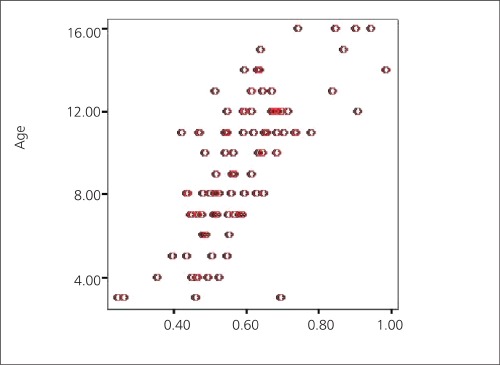
Distribution of BMD levels versus age

**Figure 2:**
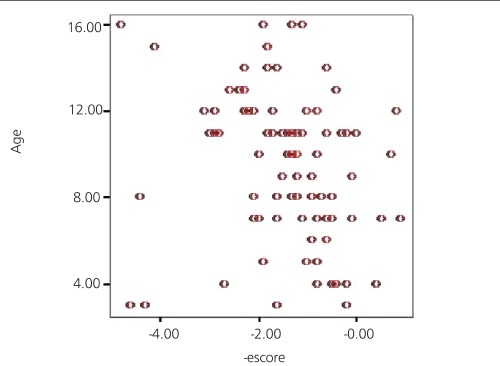
Distribution of Z-scores versus age

Mean BMD values of lumbar region and total hip region and Z-score of lumbar region in terms of whole group were 0.59±0.14 and 0.65±0.15 and -1.14±1.14, respectively. The BMD values of both lumbar spine and femur and Z-scores of lumbar spine were in correlation with age (r>0.5) (Figure 1,2). There was no correlation between BMD values and disease duration at presentation (r<0.5).

There were 25 patients (mean age: 10.8±3.2 years) with low bone mass and 66 patients (mean age: 9.5±2.6 years) had normal BMD There was a significant difference between the mean ages of the patients with low bone mass for chronological age and the patients with normal bone densitometry values (p<0.05). 

**Figure 3:**
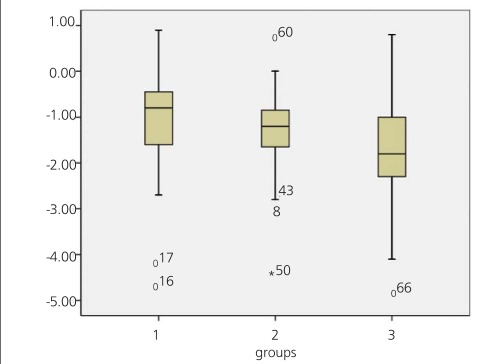
Distribution of Z-scores of three age groups

There were 27 (M/F:14/13), 36 (M/F:22/8) and 28 (M/F:13/15) patients in Group 1, Group 2 and Group 3, respectively. Mean values of lumbar and total hip BMD results of the groups were 0.48±0.09, 0.59±0.1, 0.72±0.13; 0.55±0.13, 0.67±0.12, 0.75±0.11, respectively. The mean values of vertebral Z-scores of the groups were -1.08±1.27, -1.42±1, -1.86±1.14, respectively (Figure 3). The difference between mean values of lumbar and total hip BMD results and Z-scores of the groups were statistically significant (p<0.05).

## DISCUSSION

CD causes decreased BMD at adult ages especially if the patients are untreated. Factors that cause reduced BMD are calcium malabsorption, vitamin D deficiency and inflammatory cytokines that result in destruction of bone structure (7,8). Treatment with GFD at earlier ages causes an increase in BMD (9,10). Some researchers agree that this increase is not complete (11) and disease causes an irreversible osteoporosis. The studies on adult population clearly show that GFD does not recover the effect of the disease on BMD (12). 

Since CD patients have an increased risk of osteoporosis, fracture risk was investigated in CD patients. Most researchers concluded that there was an increased risk of fracture in CD patients (13,14). However no significant difference was found between the patients with fracture compared to those without fracture in terms of Z-scores, serum calcium, 25-hydroxyvitamin D, parathormone and dietary calcium intake (13). 

Although CD is usually presented and diagnosed at early childhood (15), our patient population consists of different age groups. This observation made us compare BMD findings of various age groups. There are different studies about BMD at first diagnosis of disease showing low bone density levels in children and adult ages (16,17). Patients who receive appropriate treatment before puberty don nott become osteoporotic at adult age (18). Strict dietary therapy is needed in order to maintain normal bone density outcome (19). 

The certain age that osteopenia or osteoporosis occurs in CD patients is not clear. It was demonstrated that both asymptomatic and symptomatic CD patients had lower BMD levels at the time of diagnosis (20,21). Additionally Turner et al. showed that the younger age of CD patients at diagnosis with appropriate therapy was associated with better outcome of BMD (22). Same study mentioned that although they did not intend to assess age related changes of bone density in CD patients, they observed that there was not a significant change in bone mass for age and sex among the 29 patients involved to the study. 

In our study we included 91 patients with CD and their mean Z-scores were in a wide range (-1.14±1.14). According to our results there was not necessarily a decrease in Z-scores of CD patients at the time of diagnosis. Mean ages of patients with low bone mass were higher than patients with normal BMD. This finding led us to investigate whether there was a decreasing trend of BMD levels with increasing age groups or not. When the patients were evaluated in three groups according to age (i. e. the first group consisted of the patients in pre-school age, second was elementary school age and third was adolescent age group), there was an increase with increasing ages in terms of mean BMD. As known there is a physiological increase of BMD for growing children. Because of that the important parameter for those patients was Z-score. Although Z-scores of the patients were in a wide range, there was a decrease of Z- scores with increasing age between the groups (Group 1>Group 2>Group 3, in terms of Z-score). This means that bone mineral loss increases proportionally with age in these children.

A limitation of this retrospective study is that we compared different patients’ results. We were just aware of that the patients were on starting point without any treatment or diet. If this study is applied to the follow-up BMD results of the same patients for growing ages more meaningful results can be achieved. However in that kind of study, it could be also supposed that the other factors related to time (i.e. dietary factors, treatments etc.) influence the results. 

## CONCLUSION

Age is an important determinant of BMD levels at the time of diagnosis for CD patients. There is a decreasing trend of Z-scores. We suppose that the basal BMD levels and Z-scores of CD patients have to be stated on the earlier ages and be followed up. In this way, early diagnosis of the disease and prevention of bone mineral loss can be achieved as stated in previous reports (23).
